# Provision of a local anaesthetic minor procedures service by surgical advanced clinical practitioners: 5-year study

**DOI:** 10.1093/bjsopen/zrab073

**Published:** 2021-09-03

**Authors:** A Taib, C Hammill, A Abraham, B Fakim, P Garstang, J Carney, V Natarajan, D Subar

**Affiliations:** 1 Department of General Surgery, East Lancashire Hospitals NHS Trusts, Blackburn, UK; 2 Blackburn Research Innovation Development Group in General Surgery (BRIDGES), Blackburn, UK; 3 Women’s and Children's Division, Lancashire Teaching Hospitals NHS Foundation Trust, Preston, UK

## Abstract

**Background:**

Surgical advanced clinical practitioners (SACPs) form part of the extended surgical workforce drawn from a variety of allied healthcare backgrounds. The primary aim of this study was to determine whether there was a financial benefit in having minor surgical procedures undertaken by dedicated SACPs compared with operating lists assigned to consultant surgeons.

**Methods:**

This was a retrospective cohort study including all patients who had minor ‘lumps and bumps’ procedures undertaken between April 2014 and August 2019 at East Lancashire Hospitals NHS Trust under local anaesthetic by the general surgery team. Clinical patient information, including lesion type, was collected along with operating room staffing levels and duration of operation. The cost of the procedure was calculated as operating time multiplied by cost of staff per minute according to local banding.

**Results:**

A total of 1399 patients had a lesion excised; 907 procedures were carried out by a doctor, and the rest independently by a SACP. The majority of lesions excised were lipomas and cysts. There was no difference in the median surgical time taken between SACPs and doctors (20 (i.q.r. 14–28) min). Minor procedures carried out on consultant surgeon lists cost 62.3 per cent (€25.33) more on average than those on SACP lists (median €65.96 *versus* 40.63 respectively; *P* < 0.001).

**Conclusion:**

A dedicated and independent SACP ‘lumps and bumps’ list was financially beneficial. Operating times were similar to those of doctors. These lists safely free trainee and consultant surgeons to undertake more complex work.

## Teaser

A dedicated and independent surgical advanced care practitioner ‘lumps and bumps’ list has been shown to be a financially beneficial service. Operating times are similar to those of doctors. These lists free staff for consultant lists, potentially permitting more major procedures.

## Introduction

Implementation of the European Working Time Directive in 2008 reduced the maximum number of working hours to 48 per week. Consequently, non-medically qualified healthcare professionals have fulfilled roles previously covered by trainee surgeons during normal working hours.

This extended surgical care team includes a variety of individuals (surgical care practitioner, SCP; surgical first assistant; surgical advanced clinical practitioner, SACP). Development of the practitioner role has expanded rapidly, with the aim of improving productivity and staff capacity by spreading the service workload across more members of the surgical team^1^. According to the Royal College of Surgeons of England *A Question of Balance* report published in 2016^1^, these extended roles have been an asset to patient care, training, and efficiency. They can be deployed in a ward, clinic, or operating theatre setting.

Previous studies have explored the possible benefits and acceptability of such non-medical practitioners carrying out operative procedures. The evidence is mixed. A study^2^ exploring the impact of SCPs leading and operating in a one-stop ‘see and treat’ minor procedures clinic was positive for both surgical outcomes and patient satisfaction, whereas another^3^ highlighted a significant proportion of patients who were reluctant for an SCP to undertake their minor procedure. It has also been suggested that training the extended surgical care team is not cost-effective owing to longer than expected learning curves^4^, and there are concerns that training practitioners may take away practical opportunities from surgical trainees^5^.

The present study sought to evaluate the cost-effectiveness of non-medical practitioners carrying out minor procedures. The primary aim was to determine whether there was a financial benefit to performing minor operations on dedicated SACP ‘lumps and bumps’ lists compared with those included in elective lists allocated to consultant surgeons, not dedicated solely to minor procedures. It also aimed to compare operating times between the two groups and examine the learning curve for SACPs performing minor operations.

## Methods

This was a cohort study including all patients who had a minor ‘lumps and bumps’ procedure undertaken between April 2014 and August 2019 at East Lancashire Hospitals NHS Trust (ELHT) under local anaesthetic (LA). Patients were identified using the terms ‘skin lesions’ and ‘general surgery’ using operating theatre management software (TheatreMan Trisoft, Nottingham, UK). This included patients coded for excision of lipomas, cysts, skin lesions including neurofibromas, and other benign swellings considered suitable for LA surgery. This research was approved by the local audit committee and, as a retrospective analysis using anonymized data, patient consent was not required.

The independent variable was the profession of the chief operator: a medically qualified doctor (surgeon) on a consultant-led list containing a mix of minor and major procedures, or a SACP on an independent list dedicated to minor procedures under LA. The surgeon could be a trainee or consultant. Data extracted included patient age, sex, date of procedure, number of lesions excised, surgical time, total time in the operating room, and staff present during procedure. From this, based on a fixed average hourly rate for each grade of staff determined by local banding, the dependent variable of procedure cost was calculated. Staff costs were provided independently by the management team at ELHT in pounds sterling. Cost was then converted to euros at the exchange rate of £1 being equivalent to €1.17 on 30 June 2021.

The relationship between date and surgical time was investigated using a Pearson product-moment correlation coefficient as a proxy for the SACP learning curve.

Statistical analysis was performed using SPSS^®^ version 22.0 (IBM, Armonk, New York, USA). χ^2^ test of independence was used for categorical variables, and continuous variables with a normal or non-normal distribution were analysed using the independent-samples *t* test and Mann–Whitney *U* test respectively. *P* < 0.050 was considered statistically significant.

## Results

A database of 1402 patients was accessed and reviewed retrospectively. Three patients were excluded because of insufficient information or incorrect coding. A total of 1399 patients were included in the study, and had a lesion removed under LA by the general surgery service between April 2014 and August 2019 at ELHT. The majority of procedures (907, 64.8 per cent) were carried out by a doctor on a consultant list. Of the patients operated on by a doctor, 608 (67.0 per cent) were operated on by consultants, and the rest (299, 33.0 per cent) by surgical trainees. Some 492 patients (35.2 per cent) had procedures undertaken independently by SACPs. The lead operators in this study consisted of 19 consultants, 91 trainees, and 3 SACPs. The advanced clinical practitioners operated on a slightly older cohort of patients than their doctor colleagues (mean age 50 *versus* 47 years; *P* = 0.002). The sex ratio was similar in the two cohorts (*Table 1*).

**Table 1:** Demographic and lesion characteristics

	Consultant list	SACP list	***P*** §
	(*n* = 907)	(*n* = 492)	
Age (years)*	47(19)	50(16)	0.002^¶^
**Male patients** ^‡^	477 (52.6)	259 (52.6)	0.985
**Lesion type excised**			
Lipoma	355 (39.1)	147 (29.9)	0.001
Cyst	318 (35.1)	243 (49.4)	< 0.001
Skin lesion	102 (11.2)	68 (13.8)	0.159
> 1 lesion type	3 (0.3)	3 (0.6)	0.446
Other	129 (14.2)	31 (6.3)	< 0.001
**No. of lesions excised**†	1 (1–1)	1 (1–1)	0.869^#^
1	774 (85.3)	422 (85.8)	
2	74 (8.2)	37 (7.5)	
3	30 (3.3)	14 (2.8)	
4	6 (0.7)	4 (0.8)	
5	12 (1.3)	6 (1.2)	
≥ 6	11 (1.2)	9 (1.8)	

Values in parentheses are percentages unless indicated otherwise; values are *mean(s.d.) and †median (i.q.r.).

^‡^Data were missing for one patient. SACP, surgical advanced clinical practitioner. §χ^2^ test of independence (1.d.f.), except

^¶^independent-samples *t* test and ^#^independent-samples Mann–Whitney *U* test.

The case-mix breakdown indicated that most patients had only one type of skin lesion removed (*Table 1*). Lipomas (502, 35.8 per cent) and cysts (561, 40.1 per cent) accounted for the majority of the workload for both list types. A significantly greater proportion of patients had lipomas removed on consultant lists than on SACP lists: 355 (39.1 per cent) *versus* 147 (29.9 per cent) (*P* = 0.001). This was also the case for other skin lesions: 129 (14.2 per cent) *versus* 31 (6.3 per cent) (*P* < 0.001). Significantly more cysts were removed on SACP lists compared with consultant lists, accounting for almost half the workload: 243 (49.4 per cent) *versus* 318 (35.1 per cent) (*P* < 0.001).

There was a significantly higher number of staff present during a consultant-led list (*Table 2*). Only five SACP lists (1.0 per cent) had a surgical first assistant, whereas there was an assistant present for 124 procedures (13.7 per cent) on consultant lists (*P* < 0.001). Anaesthetists were present in approximately one-third of operations (326, 35.9 per cent) on a consultant list, whereas this was the case only for seven procedures (1.4 per cent) on an SACP list (*P* < 0.001). Although an anaesthetic practitioner was always present during a procedure, a significantly higher number were present during consultant lists (*P* < 0.001). With regards to operating room circulating personnel, two individuals were present in most procedures during consultant lists (599, 66.0 per cent) whereas it was common for only one individual to be present during SACP procedures (324, 65.9 per cent) (*P* < 0.001). There was no difference in the number of scrub nurses on each list (*P* = 0.081). In seven (1.4 per cent) of the 492 SACP procedures, a consultant was called for advice; although complications were not recorded formally, only three significant events were identified (1 haematoma, 1 stitch granuloma, 1 superficial wound dehiscence), all of which were managed conservatively.

**Table 2:** Staffing levels during procedures

Staff per procedure	Consultant list	SACP list	*P* ^†^
	(*n* = 907)	(*n* = 492)	
**Chief operator**			
1	907	492	
**Surgical assistant**			< 0.001^‡^
0	783 (86.3)	487 (99.0)	
1	124 (13.7)	5 (1.0)	
**Anaesthetist**			< 0.001^‡^
0	581 (64.1)	485 (98.6)	
1	326 (35.9)	7 (1.4)	
Anaesthetic practitioner*			< 0.001‡
1	759 (83.7)	450 (91.5)	
2	147 (16.2)	42 (8.5)	
**Scrub nurse**			0.081^§^
0	6 (0.7)	9 (1.8)	
1	849 (93.6)	461 (93.7)	
2	52 (5.7)	22 (4.5)	
Circulating staff*			< 0.001^¶^
0	8 (0.9)	26 (5.3)	
1	223 (24.6)	324 (65.9)	
2	599 (66.0)	140 (28.5)	
3	76 (8.4)	2 (0.4)	

Values in parentheses are percentages. *Data were missing for one patient. SACP, surgical advanced clinical practitioner.

^†χ^2 test of independence:

^‡^1 d.f., §2 d.f.,

^¶^3 d.f.

*Table 3* shows the cost per minute of staff during the lists. The costs of consultant surgeons and anaesthetists per minute were almost twice that of an SACP (€1.13, 1.13, and 0.59 respectively). Based on staffing levels (*Table 2*) and banding (*Table 3*), *Table 4* shows the cost of LA ‘lumps and bumps’ procedures as well as the median operating time. There was no difference in the median surgical time taken to operate on each patient. In both cohorts this was 20 (i.q.r. 14–28) min. Consultant surgeons’ operating times were marginally shorter than those of trainees: median 19 (i.q.r. 13–27) min *versus* 21 (i.q.r. 14–29) min. Minor procedures carried out on consultant surgeon lists cost 62.3 per cent (€25.33) more on average than those on SACP lists (median €65.96 *versus* 40.63; *P* < 0.001).

**Table 3:** Cost of staff per hour and minute

	Cost per h (€)	Cost per min (€)
Consultant surgeon as chief operator	67.89	1.13
SACP as chief operator	35.69	0.59
Surgical assistant	29.40	0.49
Anaesthetist	67.89	1.13
Anaesthetic nurse	20.30	0.34
Scrub nurse	20.30	0.34
Circulating staff	14.00	0.23

SACP, surgical advanced clinical practitioner.

**Table 4:** Surgical time and cost of staff per procedure

	Consultant list	SACP list	***P*** *
Surgical time (min)	20 (14–28)	20 (14, 28)	0.329
Cost of staff per procedure (**€)**	65.96 (46.95–98.78)	40.63 (30.10–53.88)	< 0.001

Values are median (i.q.r.). SACP, surgical advanced clinical practitioner. *Iindependent-samples Mann–Whitney *U* test.

**Fig. 1:**
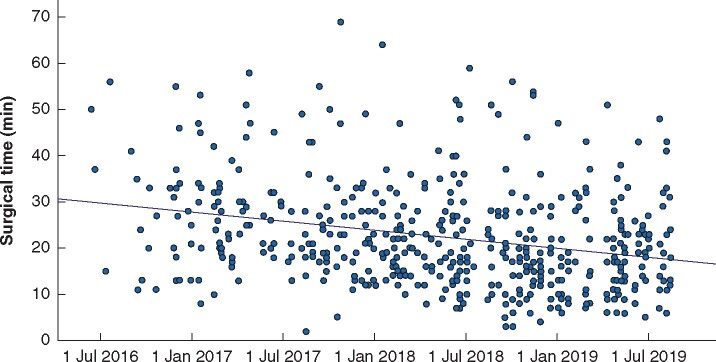
Surgical times by surgical advanced clinical practitioners throughout the study interval A negative correlation is demonstrated.

Examination of the relationship between date and operating time as an indicator of a learning curve indicated a small, negative correlation between the two variables (*r* = –0.272, *n* = 491, *P* < 0.001). The best line of fit indicated that surgical times shortened over time (*Fig. 1*).

## Discussion

This study indicated that, over a 5-year interval, there was financial benefit to SACPs performing LA procedures on independent dedicated lists compared with those undertaken on consultant surgeon-led lists. SACPs had operating times comparable to those of medically qualified doctors for such procedures.

The median cost of a minor operation on a consultant-led list was 62.3 per cent greater than when carried out by a SACP. Over the cumulative number of procedures carried out across the 5 years, this can be extrapolated to a saving of approximately €35 000. As operating times were similar for both types of list, differences in cost seem likely to reflect variation in staffing levels. Consultant-led lists were often fully staffed for a session that included major operations, with minor procedures such as ‘lumps and bumps’ using remaining sessional time.

All consultants were in hospital for the minor procedures on their lists being undertaken by trainees under direct or indirect supervision. Their cost per hour therefore remains even though they may have been carrying out other duties. The cost of an independent SACP per hour was almost half the consultant cost, so the overall savings reflected not only fewer staff present during SACP lists but also that the most expensive staff were not required routinely. In only 1.4 per cent of SACP procedures was a consultant called for advice. Although this is not included in the cost of the procedure as consultants were undertaking other activities, the limited amount of assistance is crucial if an independent SACP-led service is to be seen as generating a financial saving.

The gradual improvement in the operating times of SACPs suggested that duration of surgery was shortened by experience. Given the heterogeneity between procedures, perhaps clearer evidence of a learning curve might be better appreciated for a specific lesion.

The difference in case mix between the two lists might be a confounding factor. The preponderance of lipomas on the consultant-led lists may reflect a perception of greater difficulty requiring more time than cyst excision. Operation notes were not accessible during this study, so operative difficulty could not be assessed.

The present results of surgery undertaken by SACPs are similar to those published elsewhere^2^, when a complication rate of 2.9 per cent over 4 years was seen and patient satisfaction was high. Given the risk of significant patient harm associated with surgery, patient concerns surrounding allied healthcare professionals undertaking practical procedures have been expressed^3^, but this may be influenced by the type of surgery being offered. Training a SCP to undertake surgery that included inguinal hernia repair required an 800-hour practical training period. It was concluded that, because of the extended learning curve compared with training a doctor, it was not cost-effective^4^.

A further concern involves the impact on surgical training as minor operations have been traditionally undertaken by surgical trainees^5^. Although consultants were designated as the primary surgeon in two-thirds of the procedures, surgical trainees may not have been logged as the lead operator despite undertaking the procedure in the presence of their consultant. SACPs also provided some training for surgical trainees during their lists. Although it has been suggested that adequate supervision and training for junior doctors during practical procedures is often lacking^6^, a dedicated SACP ‘lumps and bumps’ list that provides supervision and feedback may be a worthwhile adjunct to training.

The present study has a number of limitations, including those inherent in its retrospective design. Reliance on staffing levels as the indicator of cost benefit is only part of overall cost-effectiveness based on staffing levels. Specific patient data, such as location and size of lesions, was not always available to allow quantification of the difficulty of the lesion to be excised. Patient allocation to consultant-led and SACP-led lists might be important. The present study did not consider the cost of surgical instruments and disposables. Complication rates are also key in establishing the cost-effectiveness of a SACP-led service. Postoperative complications were difficult to ascertain given the nature of the study, missing data, and the fact that minor complications might have been managed in primary care. Despite this major limitation, there was no documentation of returns to theatre for patients operated on by SACPs. There were three recorded complications of procedures on SACP lists (1 haematoma, 1 stitch granuloma, 1 superficial wound dehiscence), all of which were managed conservatively. Although these may have been underestimated, complications, the cost of antibiotics or analgesia, returns to theatre, and recurrence of lesions would all need to be evaluated to demonstrate overall cost-effectiveness.

A dedicated and independent SACP ‘lumps and bumps’ list was financially beneficial compared with minor operations being included in a consultant-led general surgical list, with similar operating times. The saving was of sufficient magnitude to indicate that a prospective study looking at cost-effectiveness in detail should be worthwhile.
